# Sleeve Gastrectomy in Patients with Type 2 Diabetes: Anthropometric and Cardiometabolic Improvements at 1, 3, 5, 7, and 9 years—Are the Initial Benefits Sustained?

**DOI:** 10.1007/s11695-024-07664-w

**Published:** 2025-03-14

**Authors:** Wahiba Elhag, Isra Elgenaied, Merilyn Lock, Walid El Ansari

**Affiliations:** 1 Hamad Medical Corporation, Doha, Qatar; 2 Weill Cornell Medicine - Qatar, Doha, Qatar; 3 Hamad Bin Khalifa University, Doha, Qatar; 4 College of Medicine, Ajman University, Ajman, United Arab Emirates; 5 Department of Surgery, Hamad Medical Corporation, Doha, Qatar

**Keywords:** Sleeve gastrectomy, Long-term outcomes, Type 2 diabetes remission, Diabetes recurrence, Weight regain

## Abstract

**Background:**

No previous study assessed the outcomes of sleeve gastrectomy (SG) beyond 5 years among adult patients with type 2 diabetes (T2DM). We appraised the evolution of 20 outcomes at 1, 3, 5, 7, and 9 years.

**Methods:**

This is a retrospective study of 361 consecutive T2DM patients who underwent SG at our institution (April 2011–December 2015). Postoperative data were compared to baseline and to previous time points using paired *t* tests. Diabetes status was also assessed at each time point.

**Results:**

The sample consisted of 65.37% females. The mean preoperative age was 44.38 ± 9.50 years and body mass index (BMI) was 43.81 ± 6.98 kg/m^2^. Generally, most improvements occurred in the first year and were sustained on the long term. Postoperatively, there was a significant and sustained weight reduction, amounting to a mean decrease of 9 kg/m^2^ in BMI at year 9 and associated excess weight loss between 59%_year 1_ and 46%_year 9_. Fasting blood glucose decreased significantly across the five time points, from 9.27 ± 4.11_preop_ to 7.06 ± 2.70_ year 9_ mmol/L. Mean HbA1c significantly decreased from 8.1%_preop_ to 6.77%_year 9_. The prevalence of complete remission of T2DM was 20.45%, 19.44%, and 20% at 5, 7, and 9 years respectively, with significant reductions in percentages of patients using diabetes medications or insulin. Between 10% and 23% of patients experienced relapse of T2DM by 5–9 years. In comparison to baseline levels, mean blood pressure, triglycerides, high-density lipoprotein, low-density lipoprotein, total cholesterol, and hepatic enzymes levels all showed improvement in the long term.

**Conclusion:**

Patients with T2DM experienced substantial improvements in most anthropometric and cardiometabolic outcomes within the first year, and these were sustained in the long term.

## Introduction

Obesity is associated with an increased risk of type 2 diabetes (T2DM), with trends in the prevalence and incidence of obesity closely mirroring those of T2DM [1]. Most patients with T2DM also suffer from obesity; hence, both conditions represent global clinical and public health challenges [2, 3]. Metabolic and bariatric and surgery (MBS) is effective for managing such patients [4, 5], achieving good glycemic and metabolic control [6].

A particularly popular MBS worldwide is sleeve gastrectomy (SG) [7], offering a variety of advantages including the apparent simplicity of the procedure, promising weight loss, glycemic and metabolic improvements, as well as other advantages [5, 8–10]. However, assessments of the durability of the improvements after SG in such patients are critical, to provide evidence about whether the initial benefits accrued are sustainable. To our knowledge, very few studies have evaluated the long-term maintenance of clinical outcomes after SG.

Indeed, few dedicated studies have appraised the effects of SG among patients with T2DM [11–15]. In addition, these studies have often been limited by small sample sizes [11, 13, 16], restricted follow up durations of up to 5 years [11, 13–16], non-reporting of outcomes at each time point throughout the study period [12, 14, 16], a lack of consideration of changes in diabetes status (complete/partial remission, improvement, unchanged, relapse) [11, 14, 16], and lack of appraisal of the wide range of metabolic factors to including lipid profiles and liver enzymes [11, 13–16].

Therefore, the aim of the current study was to assess the long-term evolution of 20 characteristics at 1, 3, 5, 7, and 9 years after SG among adults with T2DM. The specific objectives were to appraise changes in anthropometric, clinical, glycemic, lipid, and hepatic characteristics at these five time points, compared to preoperative values. The study also assessed changes in diabetes status at each of these time points.

## Materials and Methods

### Study Design, Ethics, and Participants

**Fig. 1:**
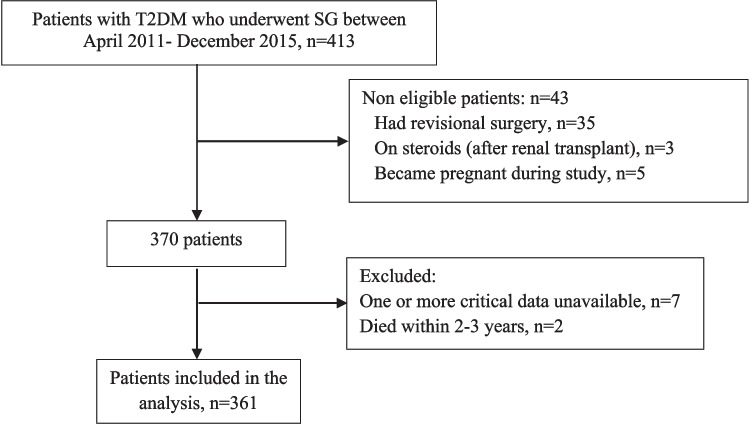
Patient flow chart. *T2DM* type 2 diabetes mellitus; *SG* sleeve gastrectomy

The ethics committee at the Medical Research Center at our institution approved this retrospective study (Protocol No. 16169/16). We included all consecutive patients who underwent SG between April 2011 and December 2015 at the MBS Centre at our institution. Inclusion criteria were patients aged 18–65 years with T2DM and a body mass index (BMI) ≥ 35 kg/m^2^. Exclusion criteria included patients with type 1 diabetes, those who underwent revisional SG, who became pregnant during the study period, or who were on steroids (e.g., renal transplant patients). Figure 1 shows the flow chart of the 361 patients included in the final analysis. As many patients are not “permanently” lost to follow-up, i.e., might miss specific appointments but show up again for subsequent scheduled visits, the tables depicted in the results section report the number of patients included in each analysis based on the variable and time point analyzed.

### Data Collection

Using the hospital databases, patients’ electronic records were searched at the preoperative (preop) time point as well as at 1, 3, 5, 7, and 9 years after SG. We retrieved data that included two demographic (age, sex), two anthropometric (weight, height), four clinical [systolic/diastolic blood pressure (SBP, DBP), number of patients on diabetes medications, type of diabetes medication/s, number of patients on insulin therapy], five glycemic [fasting blood glucose (FBG), hemoglobin A1c (HbA1c), insulin level, C-peptide] and six lipid and hepatic [low density lipoprotein (LDL), high density lipoprotein (HDL), total cholesterol (TC), triglyceride (TG), alanine aminotransferase (ALT), aspartate aminotransferase (AST)] variables.

### Definitions

Weight regain was defined as an increase in body mass of ≥ 10 kg from nadir weight, and nadir weight was defined as lowest measured post-operative body weight [17, 18]. We used the standardized American Society of Bariatric and Metabolic Surgery definitions of evolution of T2DM after MBS (complete/partial remission, improvement, unchanged, relapse) [19], in line with previous studies [4, 5].

### Data Processing and Statistical Analysis

BMI and insulin resistance [homeostasis model assessment of insulin resistance (HOMA-IR)] were calculated using methods described previously [20, 21]. Percentage of excess weight lost (EWL%) and total weight lost (TWL%) were calculated using standard formulae [19]. Weight regain and nadir weight were identified using the logic test function in Microsoft Excel.

All statistical analyses were undertaken in SPSS (version 29.0, IBM Corp.). Descriptive statistics for continuous variables were presented as means and standard deviations, and those for categorical variables were presented as frequency of cases and percentage of the whole sample at baseline (preop). Changes in all variables were tested against pre-surgical values using paired-samples *t* tests of the patients who had available measures at both time points.

Descriptive data from the paired-samples *t* tests were presented in tables for each post-operative time point. In cases where outcomes substantially deviated from a normal distribution, data were presented as median with 25th and 75th percentiles and were statistically tested against baseline using Wilcoxon signed-rank tests. Differences in all change variables (i.e., the prevalence of weight regain, EWL%, and TWL%) were calculated from the earliest possible time point and statistically tested relative to the time point immediately prior. The significance level for all statistical tests was set at *P* = 0.05.

## Results

### Preoperative Characteristics

The sample’s mean age was 44.38 ± 9.50 years, with roughly two-thirds females (65.37%) (Table 1). Preoperatively, associated medical conditions included hypertension and dyslipidemia (51.86% and 44.80% respectively). Mean T2DM duration was 8.78 ± 7.08 years, and 63.8% of patients had diabetes for ≥ 5 years. Across the sample, 57.06% were receiving diabetic medication/s, 27.25% were on insulin therapy, and 22.32% were on both.

**Table 1:** Pre-operative characteristics of the sample

Characteristic	Value
Age (years, M ± SD)	44.38 ± 9.50
Height (meter, M ± SD)	1.64 ± 0.10
Sex	
Female	236 (65.37)
Male	125 (34.63)
Hypertension	181 (51.86)
Dyslipidemia	155 (44.80)
Diabetes duration (years, M ± SD)	8.78 ± 7.08
< 5	79 (36.2)
$\ge$5	139 (63.8)
Treatment modality^a^ *N* (%)	
Insulin therapy	94 (27.25)
Diabetes medication/s	206 (57.06)
Diabetes medication + insulin	77 (22.32)
Type of diabetes medication^a^	
GLP-1 receptor agonists	68 (19.10)
Insulin sensitizers^b^	150 (41.55)
Sulfonylureas	89 (25.72)
HbA1c *N* (%)	
≤ 6.5	86 (24.50)
> 6.5–8	105 (29.91)
> 8–9	54 (15.38)
> 9	106 (30.20)

Cell values represent frequency (percent) unless otherwise stated, *M* mean, *SD* standard deviation, *GLP 1* glucagon-like peptide 1. ^a^Percentages do not add to 100% due to patients using one or more medication/s, or not taking any medication at all, ^b^Includes biguanides

### Anthropometric, Clinical, Glycemic, Lipid, and Hepatic Changes Across Time

**Fig. 2:**
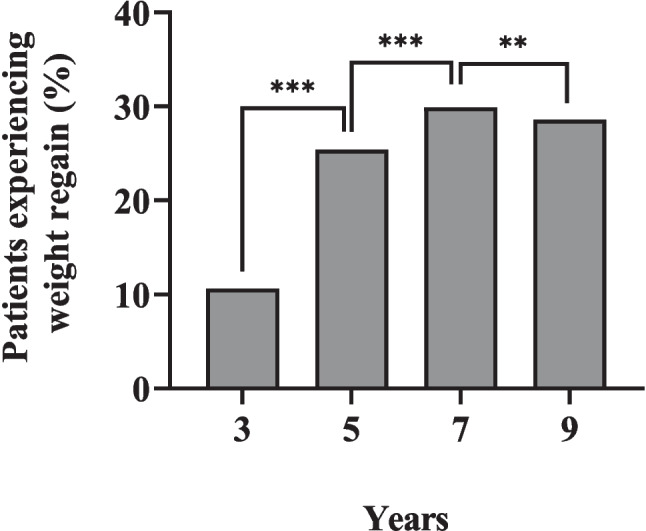
Patients with weight regain at 3, 5, 7 and 9 years after sleeve gastrectomy, and their comparisons with previous time points Significant change from the previous timepoint at the level of **P < 0.01, *** *P* < 0.001

Table 2 shows the short-, mid-, and long-term anthropometric, clinical, glycemic, lipid, and hepatic changes. Across all five time points, weight and BMI were consistently and significantly lower than their preop values (*p* < 0.001 for all time points), reflected by EWL% ranging between 45.80 ± 20.57 and 59.34 ± 26.03%. Weight regain started at year 3, with an increasing prevalence between 10.6% and 29.9% across the four time points (Fig. 2).

**Table 2:** Patient characteristics across the study period

Variable		Pre-op	Year 1	Year 3	Year 5	Year 7	Year 9	
Anthropometric								
Weight (kg)	M ± SD	117.41 ± 21.07	87.26 ± 16.04	90.39 ± 17.25	92.16 ± 17.58	97.96 ± 20.14		98.06 ± 17.12
	*n*	361	257	311	314	93		33
	*P*	**—**	< *0.001*	< *0.001*	*0.001*	< *0.001*		< *0.001*
BMI (kg/m^2^)	M ± SD	43.81 ± 6.98	33.06 ± 6.17	34.02 ± 6.35	43.91 ± 7.03	36.06 ± 6.47		35.75 ± 5.81
	*n*	361	257	311	314	93		33
	*P*	**—**	< *0.001*	< *0.001*	< *0.001*	< *0.001*		< *0.001*
EWL%	M ± SD	**—**	59.34 ± 26.03	54.10 ± 26.32	49.70 ± 26.46	45.80 ± 20.57		45.83 ± 20.74
	*n*	361	257	248	279	77		28
	*P*	**—**	**—**	*0.001*	< *0.001*	0.244		0.616
TWL%	M ± SD	**—**	23.73 ± 9.67	21.67 ± 10.27	20.21 ± 10.64	19.26 ± 9.04		19.67 ± 9.04
	*n*	**—**	257	248	279	77		28
	*P*	**—**	**—**	< *0.001*	< *0.001*	0.153		0.373
Clinical								
SBP (mmHg)	M ± SD	131.99 ± 13.84	125.22 ± 13.60	124.45 ± 16.04	124.22 ± 14.32	127.24 ± 17.72		127.94 ± 27.25
	*n*	258	188	231	243	67		17
	*P*	**—**	< 0.001	< 0.001	< 0.001	0.099		0.686
DBP (mmHg)	M ± SD	77.01 ± 10.48	73.93 ± 9.75	74.68 ± 9.86	73.98 ± 9.83	73.71 ± 10.53		77.82 ± 12.10
	*n*	257	188	231	242	66		17
	*P*	**—**	*0.001*	*0.010*	< *0.001*	*0.043*		0.927
Glycemic								
FBG (mmol/l)	M ± SD	9.27 ± 4.11	6.03 ± 1.96	6.78 ± 3.07	6.89 ± 2.82	6.98 ± 3.06		7.06 ± 2.70
	*n*	337	290	260	260	82		23
	*P*	**—**	< *0.001*	< *0.001*	< *0.001*	< *0.001*		*0.006*
HbA1c (%)	M ± SD	8.14 ± 1.93	6.38 ± 1.37	6.62 ± 1.70	6.86 ± 1.70	6.63 ± 1.44		6.77 ± 1.29
	*n*	351	291	276	283	91		28
	*P*	**—**	< *0.001*	< *0.001*	< *0.001*	< *0.001*		< *0.001*
Insulin level (μ/dl)	M ± SD	18.39 ± 14.98	12.96 ± 8.27	14.59 ± 5.80	12.56 ± 12.12	13.88 ± 12.02		12.97 ± 3.17
	*n*	70	11	8	7	12		3
	*P*	**—**	0.151	0.238	*0.016*	UTC		UTC
C-Peptide (ng/dl)	M ± SD	3.19 ± 1.92	2.29 ± 1.39	2.18 ± 0.93	3.20 ± 1.29	2.25 ± 1.21		2.29 ± 0.37
	*n*	176	42	25	16	14		4
	*P*	**—**	0.075	*0.027*	0.536	UTC		UTC
HOMA-IR	M ± SD	6.75 ± 5.20	5.14 ± 5.12	4.42 ± 2.47	4.45 ± 3.12	4.36 ± 3.00		3.91 ± 1.56
	*n*	67	9	8	5	10		12
	*P*	**—**	0.194	0.433	0.159	UTC		UTC
Lipid								
LDL (mmol/l)	M ± SD	3.07 ± 1.67	3.23 ± 1.14	2.94 ± 0.98	2.80 ± 0.95	2.83 ± 1.16		2.77 ± 1.08
	*n*	308	232	220	216	61		23
	*P*	**—**	*0.031*	0.761	*0.011*	0.416		0.220
HDL (mmol/l)	M ± SD	1.16 ± 0.32	1.34 ± 0.35	1.41 ± 0.37	1.41 ± 0.36	1.42 ± 0.39		1.35 ± 0.37
	*n*	302	226	215	211	55		21
	*P*	**—**	< *0.001*	< *0.001*	< *0.001*	< *0.001*		0.245
TC (mmol/l)	M ± SD	4.98 ± 1.05	5.13 ± 0.98	4.96 ± 1.26	4.82 ± 1.05	4.93 ± 1.24		4.72 ± 1.16
	*n*	314	240	228	223	64		24
	*P*	**—**	*0.008*	0.450	*0.024*	0.641		0.050
TG (mmol/l)	M ± SD	2.02 ± 1.59	1.30 ± 0.64	1.33 ± 0.80	1.33 ± 0.71	1.39 ± 0.75		1.43 ± 0.71
	*n*	306	233	219	213	55		21
	*P*	**—**	< *0.001*	< *0.001*	< *0.001*	< *0.001*		*0.047*
Hepatic								
ALT (U/L)	M ± SD	35.56 ± 27.33	17.03 ± 10.90	17.77 ± 15.36	19.71 ± 21.34	19.84 ± 13.04		17.45 ± 6.32
	*n*	294	217	218	215	60		18
	*P*	**—**	< *0.001*	< *0.001*	< *0.001*	< *0.001*		< *0.001*
AST (U/L)	M ± SD	27.81 ± 18.73	17.29 ± 7.09	19.05 ± 21.22	19.13 ± 11.48	19.33 ± 6.49		20.17 ± 5.73
	*n*	292	212	214	211	54		18
	*P*	**—**	< *0.001*	< *0.001*	< *0.001*	< *0.001*		*0.029*

*Pre-op* pre-operative, *BMI* body mass index, *EWL%* excess weight loss percent, *TWL%* total weight loss percent, *SBP* systolic blood pressure, *DBP* diastolic blood pressure, *HOMA-IR* homeostasis model assessment of insulin resistance, *TC* total cholesterol, *TG* triglycerides, *ALT* alanine aminotransferase, *AST* aspartate aminotransferase, *P* significance of the comparison of given timepoint vs preoperative level, *UTC* unable to compute, Italicized cells indicate statistical significance

**Fig. 3:**
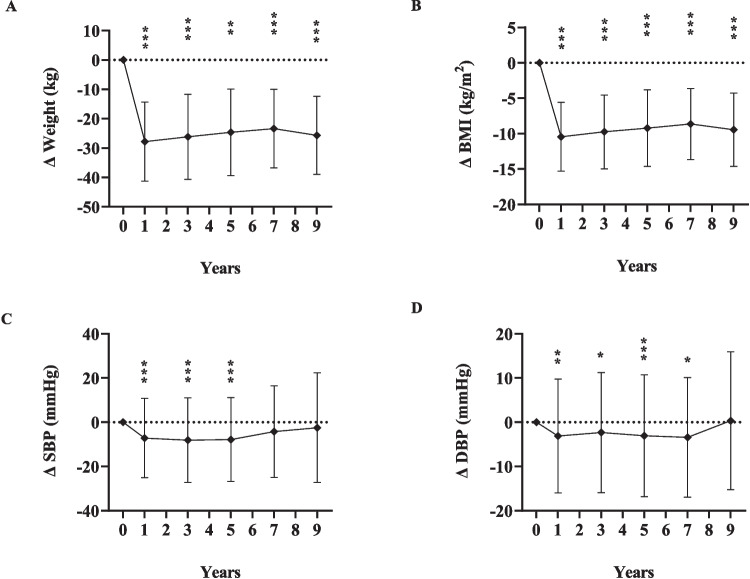
Changes (Δ) in anthropometric and clinical measures at 1, 3, 5, 7, and 9 years after sleeve gastrectomy, relative to baseline *BMI* body mass index; *SBP* systolic blood pressure; *DBP* diastolic blood pressure; diamonds indicate the mean change value (Δ); error bars indicate standard deviations of the change value (*σ*_Δ_); dotted line represents zero change from baseline; significant change from baseline at the level of *P < 0.05; ** *P* < 0.01; ***P < 0.001

Weight and BMI showed significant reduction at each time point when compared to baseline measurements (Fig. 3 A and B). SBP and DBP decreased significantly compared to baseline, the former across three time points (years 1–5), the latter across four time points (years 1–7) (Table 2), and their long-term changes (Δ) are depicted in Fig. 3 C and D.

Table 2 also shows that FBG and HbA1c improvements occurred in the first year and were sustained thereafter. Mean FBG was significantly reduced relative to baseline, across the five time points from 9.27 ± 4.11_preop_ to 7.06 ± 2.70_ year 9_ mmol/l. HbA1c significantly improved from 8.1% _preop_ to its lowest level of 6.4%_year 1_, to reach 6.8% at year 9. Insulin level and C-peptide both decreased compared to preop levels, although reduction of the former was significant only at year 5 and the latter only at year 3*.*

Table 2 further depicts that TGs were significantly lower than baseline at all post-surgical time points, whilst HDL significantly increased at four time points (years 1, 3, 5, 7) compared to preop values. Relative to baseline, LDL and TC improved through years 3–9, but this was significant only in year 5 (*P* = 0.011 and *P* = 0.024 respectively)*.* AST and ALT significantly improved across all time points compared to preop levels (*P* < 0.001 for all).

**Fig. 4:**
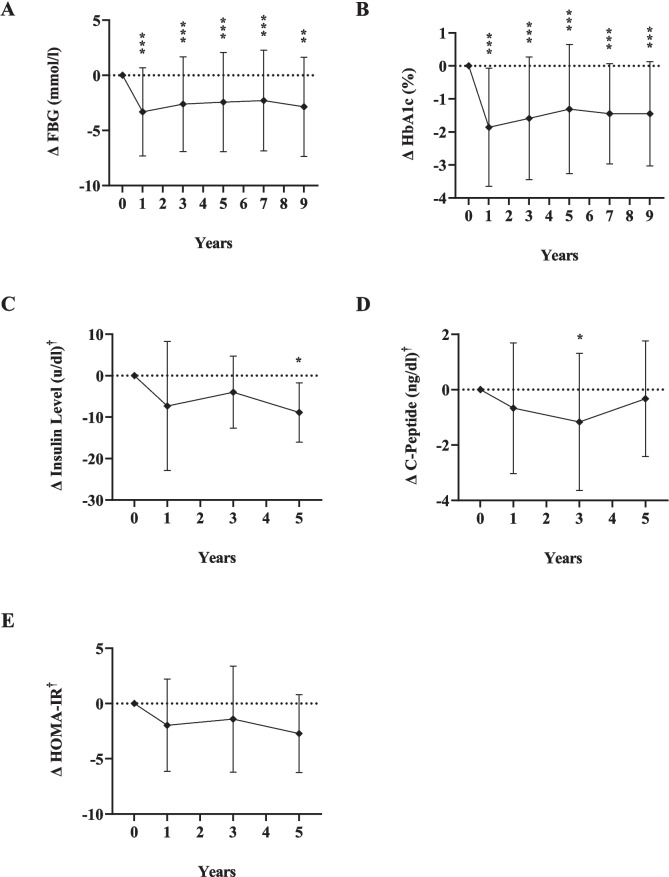
Changes (Δ) in glycemic measures at 1, 3, 5, 7, and 9 years after sleeve gastrectomy, relative to baseline *FBG* fasting blood glucose; *HbA1c* hemoglobin A1c; *HOMA IR* homeostasis model assessment of insulin resistance; diamonds indicate the mean change value (Δ); error bars indicate standard deviations of the change value (*σ*_Δ_); dotted line represents zero change from baseline; *significant change from baseline, *P* < 0.05; **P < 0.01; ***P < 0.001. For insulin level, C-peptide, and HOMA-IR, statistical tests not run for comparisons of years 5–7 and years 7–9 due to insufficient patients with measures at both time points

**Fig. 5:**
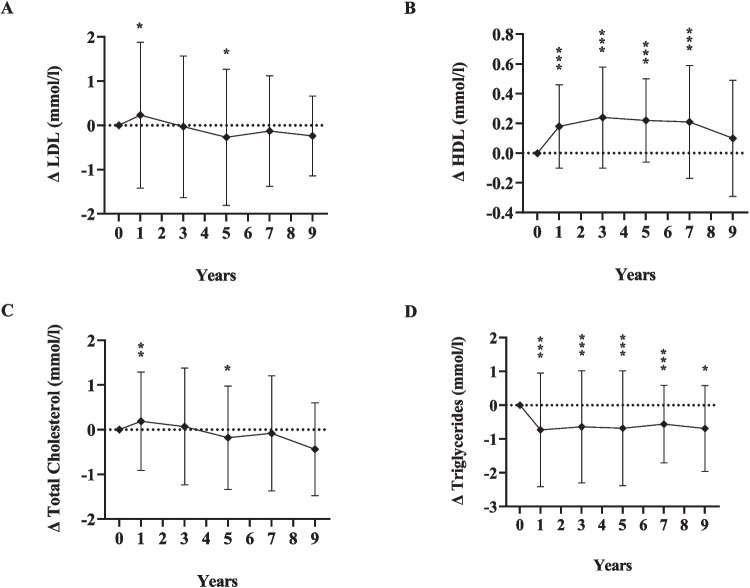
Changes (Δ) in lipid measures at 1, 3, 5, 7, and 9 years after sleeve gastrectomy, relative to baseline *LDL* low-density lipoprotein; *HDL* high-density lipoprotein; diamonds indicate the mean change value (Δ); error bars indicate standard deviations of the change value (*σ*_Δ_); dotted line represents zero change from baseline; significant change from baseline at the level of *P < 0.05; **P < 0.01; *** *P* < 0.001

**Fig. 6:**
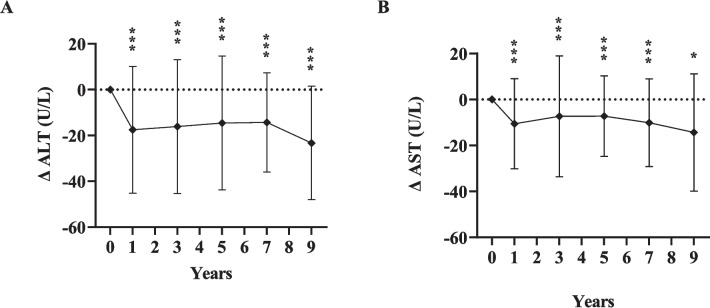
Changes (Δ) in hepatic measures at 1, 3, 5, 7, and 9 years after sleeve gastrectomy, relative to baseline *ALT* alanine aminotransferase; *AST* aspartate aminotransferase; diamonds indicate the mean change value (Δ); error bars indicate standard deviations of the change value (*σ*_Δ_); dotted line represents zero change from baseline; significant change from baseline at the level of *P < 0.05; **P < 0.01; ***P < 0.001

The long-term changes (Δ) of the variables at each time point relative to baseline are illustrated in Figs. 4, 5, and 6.

### Evolution of Diabetes Therapy Across Time

**Fig. 7:**
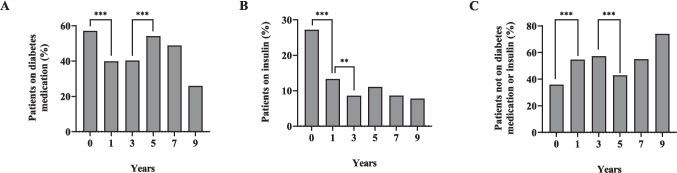
Percentage of patients on **A** diabetes medication; **B** insulin therapy; **C** not on diabetes medication or insulin, comparisons with previous time points Significant change from previous timepoint at level of **P < 0.01; ***P < 0.001

Table 3 shows that relative to preop values, the mean proportion of patients on insulin therapy, and those on diabetes medication were both significantly reduced over time, reaching approximately one third and one half respectively by year 9. Similarly, the median number of diabetes medication was also significantly decreased. The mean proportion of patients not requiring any form of diabetes therapy significantly increased from 35.92%_preop_ to 68.18%_year 9_. Figure 7 shows the percentage of patients on diabetes medication, on insulin therapy, or those not requiring any medication after SG, with each time point compared to the *previous* time point.

**Table 3:** Evolution of diabetes therapy across the study period

Variable		Pre-op	Year 1	Year 3	Year 5	Year 7	Year 9
Insulin therapy	*N* (%)	94 (27.20)	45 (13.30)	29 (8.80)	37 (11.00)	11 (9.00)	6 (9.70)
	*n*	345	338	331	337	122	62
	*P*	**—**	< *0.001*	< *0.001*	< *0.001*	< *0.001*	*0.003*
Diabetes medication	*N* (%)	206 (57.06)	141 (39.94)	138 (40.2)	184 (52.9)	59 (42.8)	21 (26.6)
	*n*	361	353	343	348	138	79
	*P*	**—**	< *0.001*	< *0.001*	0.127	0.111	*0.014*
Number of diabetes medication/s	Median	1.00	0.00	1.00	1.00	0.00	0.00
	(25, 75)^a^	(0.00, 1.00)	(0.00, 1.00)	(0.00, 1.00)	(0.00, 2.00)	(0.00, 2.00)	(0.00, 1.00)
	*n*	361	353	343	348	138	79
	*P*	**—**	< *0.001*	< *0.001*	0.341	*0.005*	0.673
Not on diabetes medication or insulin	*N* (%)	125 (35.92)	186 (54.71)	146 (43.84)	147 (43.24)	67 (53.60)	45 (68.18)
	*n*	348	340	333	340	125	66
	*P*	**—**	< *0.001*	< *0.001*	*0.020*	*0.006*	*0.001*

^a^25^th^ and 75th percentile, *N* number of patients with given outcome of interest (nominator), *n* total number of patients for the given analysis (denominator),* P* significance of the comparison of given timepoint vs preoperative level, Italicized cells indicate statistical significance

### Evolution of Diabetes Status Across Time Points

The prevalence of complete remission was between 36.5%_year 1_ and 20%_year 9_ (Table 4), and partial remission was ≈ 8–10% of patients across the five time points. The percentages of patients with improvements in T2DM but not meeting the criteria for full or partial remission were 47% _year 1_, 32% _year 3_, 35% _year 5_, 42% _year 7_, and 50% _year 9_. Conversely, no change in T2DM status was observed in 9.7–11% of patients over the long term (years 5–9), and relapse was first noted in 12.24%_year 3_, then ranged between 23%_year 5_ and 10%_year 9_.

**Table 4:** Evolution of diabetes across five time points

Diabetes status^a^	Year 1 *n* = 304	Year 3 *n* = 294	Year 5 *n* = 269	Year 7 *n* = 72	Year 9 *n* = 10
Complete remission	111 (36.51)	100 (34.01)	55 (20.45)	14 (19.44)	2 (20.00)
Partial remission	25 (8.22)	32 (10.88)	27 (10.04)	6 (8.33)	1 (10.00)
Improvement	143 (47.04)	97 (32.99)	95 (35.32)	30 (41.67)	5 (50.00)
Unchanged	25 (8.22)	29 (9.86)	30 (11.15)	7 (9.72)	1 (10.00)
Relapse	**—**	36 (12.24)	62 (23.05)	15 (20.83)	1 (10.00)

Cell values represent frequency (percent) [*n* (%)]; only patients with complete available information included in the analysis; — not applicable; ^a^Adopted from [19]

## Discussion

The long-term metabolic effects of SG among diabetic patients beyond 5 years remain largely unknown. The current study is the largest series to date, with 9 years of follow up. We evaluated 20 anthropometric, clinical, glycemic, lipid, and hepatic variables, as well as changes in diabetes status, to provide important knowledge about the sustainability of clinical improvements after SG among patients with T2DM.

Our main findings indicate that, overall, patients with T2DM who underwent SG experienced substantial long-term improvements across several parameters comprising weight reduction, enhanced glycemic, and improved metabolic profiles. In addition, there were long-term improvements in diabetes status, evidenced by a decrease in the number of patients requiring diabetes medications or insulin therapy, increased number of patients meeting the criteria for remission and partial remission, and the general improvements in glycemic measures.

Weight loss was significant and sustained, translating into a mean decrease of 9 kg/m^2^ in BMI at year 9. Such improvements were mirrored by EWL% ranging between 59%_year 1_ and 46%_year 9_. Our findings are consistent with those of previous studies, including one that followed patients with T2DM for a median of 6 years and noted significant long-term BMI reduction (8 kg/m^2^), with EWL% of 43% [12]. Similarly, a meta-analysis reported EWL% of 49.5% and BMI reduction of 10.7 kg/m^2^ at 5 years after SG [22]. Collectively, the findings of the current study indicate that these improvements can be sustained up to 9 years post-surgery. The proposed etiologies for the improvement in cardiometabolic parameters observed after SG include reduced ghrelin levels, favorable alterations in bile acids, and enhanced incretin [23].

As with all MBS, weight regain is not uncommon after SG [18]. We observed a 10.6% weight regain at year 3, increasing to 29.9% at the four subsequent time points. However, these results are relatively favorable when compared to studies from China and the USA where long-term weight regain following SG among patients with T2DM has been documented to range between 29.5% and 40% [12, 13]. Weight regain has a complex etiology that includes hormonal, metabolic, dietary, behavioral, psychological, and anatomical surgical factors [17, 24]; hence, regular follow ups with a multidisciplinary team are essential to address these underlying factors and optimize the long-term maintenance of weight loss [25].

Over the course of the study, we observed substantial improvements in glycemic control, evidenced by significant reductions in FBG and HbA1c. Mean FBG decreased significantly from 9.27_preop_ to 7.06_ year 9_ mmol/L. Others have reported similar findings among patients with T2DM 5 years after SG [11, 15, 16]. Our cohort also displayed a significant decrease in HbA1c from 8.1%_preop_ to 6.9%_year 9_. In agreement, two previous randomized controlled trials among patients with T2DM comparing the long-term outcomes of medical management versus surgical intervention (including SG) demonstrated significant reductions in HbA1c from 8.7%_baseline_ to 7.2%_year 7_ [26] and from 9.5%_baseline_ to 7.1%_year 5 _[27]. In conjunction with improved glycemic control, we noted a marked reduction in the proportion of patients requiring diabetes medication or insulin therapy. There was also a notable increase in the number of patients no longer requiring any diabetes medication, along with a substantial decrease in the overall number of diabetes medications used by patients. These findings are consistent with the literature where the percentage of patients with T2DM requiring insulin decreased from 42%_baseline_ to 25%_year 5_ after SG [12], with significant reductions in the number of diabetes medications used [27, 28].

We examined five levels of change in T2DM status (complete remission, partial remission, improvement, unchanged, recurrence) [19]. Complete remission of T2DM ranged between 19.44 and 20.45% at 5–9 years in the current series. The literature reveals wide variations of complete remission of T2DM ranging from 9% and 80% five years after SG [12, 13, 28–30]. Such variations may be due to the use of different criteria for defining complete remission, rendering it difficult to directly compare data across studies. Partial remission after SG has previously been reported by only few studies with rates ranging from 5.9% to 15% [12, 13, 15, 30, 31]. This is in agreement with our 8.33%–10% partial remission rate at 5–7 years. As for T2DM improvement, 35.3%–50% of our cohort improved on the long term, falling within the 10%–52% improvement rates reported by others at five years post-SG [12, 15, 28]. Likewise, our 8.22%–11.15% unchanged T2DM status is also consistent with the 17%–27% rates observed elsewhere [12, 28]. Improvements in glycemic control after SG can be attributed to the decreased insulin resistance, enhanced GLP release, and improved beta cell function [32].

Approximately 10–23% of our T2DM patients experienced relapse on the long term (5–9 years). Late relapse can occur after MBS, with varying incidence rate based on the definition of relapse used, patient population, type of surgery, as well as duration and completeness of follow-up [12]. These factors might collectively explain the wide variations in the relapse rates reported after SG, ranging between 5.9% to 38% [13, 15, 28, 33]. Despite relapse, studies have shown that such patients still displayed significant improvements in their metabolic profile, comparable to those of patients with long-term remission, suggesting that T2DM relapse years after SG should not be considered a failure [4, 34]. Overall, MBS effectively alters the trajectory of cardiometabolic risk factors, providing lasting benefits.

Likewise, mean SBP and DBP showed significant reductions compared to pre-surgery levels, which was maintained over 5–7 years. Others have reported similar findings [12, 28]. While weight loss is the primary factor in improving blood pressure, other contributing factors include the effects of gut hormones, enhanced insulin sensitivity, reduced arterial stiffness, and decreased activation of the renin–angiotensin–aldosterone system [35]. Similarly, in the current study, TG decreased across all time points whilst HDL significantly increased across four time points. Interestingly, improvements were less striking for LDL and TC. Consistent with our findings, others observed favorable HDL and TG changes after LSG, but no significant effect on LDL and TC [12, 27].

Furthermore, the current study demonstrated significant improvements in AST and ALT levels at all time points compared to preoperative values, suggesting that SG could result in improvement of fatty liver disease. Previous research assessing the efficacy of SG on the remission of non-alcoholic fatty liver disease among patients with T2DM have reported improvement of liver function tests, where serum liver enzymes significantly decreased 1–3 years after surgery [36]. While weight loss alone can contribute to improvements in liver enzymes, weight loss–independent mechanisms have also been postulated. These include post-operative bile acid, enterohepatic circulation changes, and gut microbiota–mediated mechanisms [36, 37].

Despite the positive findings outlined above, this study has limitations. Retrospective designs have their inherent limitations in terms of data completeness and quality. For a broader perspective, the inclusion of outcomes such as quality of life, general health perceptions, and T2DM complications (e.g., retinopathy, nephropathy) would have been insightful. A larger sample size would have enhanced the study’s power to detect significant differences particularly at the 7- and 9-year follow-ups. About 20% of patients used GLP-1 receptor agonists, which have dual effects on weight and glucose management, raising the question of whether the sustained effects on T2DM remission and weight control in later years could be partially attributed to GLP-1 effect. Future research would benefit to address these points. Despite this, to our knowledge, the study is the largest series with the longest follow-up time to date, assessing outcomes up to 9 years, across a wide range of critical variables. We reported changes at five distinct post-operative time points, providing a life course longitudinal profile, and capturing the fine changes, the sustainability of improvements, and the remission of associated medical conditions, in contrast to studies that reported cumulative average changes over a mean follow-up period [12]. We employed the American Society of Bariatric and Metabolic Surgery definitions to categorize and detect changes in five levels of diabetes status (complete remission, partial remission, improvement, unchanged, relapse), allowing a nuanced classification of patients, compared to other studies [11, 13, 14].

## Conclusion

The findings of this study add new and important insights into the longevity of the benefits after SG among patients with T2DM. Marked improvements occurred in the first year and were sustained on the long term, with significantly enhanced anthropometric, glycemic, lipid, and hepatic profiles. Patients also experienced long-term improvements in diabetes status and glycemic control, with reductions in the usage of both diabetes medications and insulin. The study also confirms that the initial benefits of SG are largely sustainable for up to 9 years among the majority of T2DM patients. Such knowledge may help inform the reduction of obesity-related health risks, overall disease burden, and cardiovascular risk related to T2DM.

## Data Availability

Data could be available upon reasonable request, and the agreement of the institution where the research was implemented.
